# Comprehensive regulatory networks for tomato organ development based on the genome and RNAome of MicroTom tomato

**DOI:** 10.1093/hr/uhad147

**Published:** 2023-07-19

**Authors:** Jia-Yu Xue, Hai-Yun Fan, Zhen Zeng, Yu-Han Zhou, Shuai-Ya Hu, Sai-Xi Li, Ying-Juan Cheng, Xiang-Ru Meng, Fei Chen, Zhu-Qing Shao, Yves Van de Peer

**Affiliations:** College of Horticulture, Academy for Advanced Interdisciplinary Studies, Nanjing Agricultural University, Nanjing 210095, China; College of Horticulture, Academy for Advanced Interdisciplinary Studies, Nanjing Agricultural University, Nanjing 210095, China; State Key Laboratory of Pharmaceutical Biotechnology, School of Life Sciences, Nanjing University, Nanjing 210023, China; College of Horticulture, Academy for Advanced Interdisciplinary Studies, Nanjing Agricultural University, Nanjing 210095, China; College of Horticulture, Academy for Advanced Interdisciplinary Studies, Nanjing Agricultural University, Nanjing 210095, China; State Key Laboratory of Pharmaceutical Biotechnology, School of Life Sciences, Nanjing University, Nanjing 210023, China; College of Horticulture, Academy for Advanced Interdisciplinary Studies, Nanjing Agricultural University, Nanjing 210095, China; College of Horticulture, Academy for Advanced Interdisciplinary Studies, Nanjing Agricultural University, Nanjing 210095, China; College of Tropical Crops, Sanya Nanfan Research Institute, Hainan University, Haikou 570228, China; Hainan Yazhou Bay Seed Laboratory, Sanya 572025, China; State Key Laboratory of Pharmaceutical Biotechnology, School of Life Sciences, Nanjing University, Nanjing 210023, China; College of Horticulture, Academy for Advanced Interdisciplinary Studies, Nanjing Agricultural University, Nanjing 210095, China; Department of Plant Biotechnology and Bioinformatics, VIB-UGent Center for Plant Systems Biology, Ghent University, B-9052 Ghent, Belgium; Department of Biochemistry, Genetics and Microbiology, University of Pretoria, Pretoria 0028, South Africa

## Abstract

MicroTom has a short growth cycle and high transformation efficiency, and is a prospective model plant for studying organ development, metabolism, and plant–microbe interactions. Here, with a newly assembled reference genome for this tomato cultivar and abundant RNA-seq data derived from tissues of different organs/developmental stages/treatments, we constructed multiple gene co-expression networks, which will provide valuable clues for the identification of important genes involved in diverse regulatory pathways during plant growth, e.g. arbuscular mycorrhizal symbiosis and fruit development. Additionally, non-coding RNAs, including miRNAs, lncRNAs, and circRNAs were also identified, together with their potential targets. Interacting networks between different types of non-coding RNAs (miRNA-lncRNA), and non-coding RNAs and genes (miRNA-mRNA and lncRNA-mRNA) were constructed as well. Our results and data will provide valuable information for the study of organ differentiation and development of this important fruit. Lastly, we established a database (http://eplant.njau.edu.cn/microTomBase/) with genomic and transcriptomic data, as well as details of gene co-expression and interacting networks on MicroTom, and this database should be of great value to those who want to adopt MicroTom as a model plant for research.

## Introduction

Tomato (*Solanum lycopersicum*) is one of the most popular fruits (although usually referred to as a vegetable) in the world. In 2020, its annual worldwide production was estimated at ~186 millions of tons and has been increasing every year (http://www.fao.org/faostat). Tomato is also an emerging model plant system for developmental biology and, in some cases, can be an better option than *Arabidopsis thaliana*, e.g. for studies of fruit development [1, 2], metabolism [3–5], plant–pathogen interactions [6, 7], and arbuscular mycorrhizal (AM) symbiosis [8, 9].

MicroTom is a tomato cultivar and currently a widely applied experimental model plant for laboratory studies. This cultivar has a smaller size than regular tomato cultivars (e.g. Heinz 1706 and M82), which, together with a shorter growth cycle and higher transformation efficiency, makes it one of the best choices for a laboratory model among tomato cultivars. However, a high-quality genome has been lacking for this model cultivar, despite the fact that hundreds of tomato cultivars/accessions have been (re-)sequenced already [4, 10–12]. Nowadays, developmental biologists rely on the Heinz tomato genome as a reference, whereas functional experiments are mainly performed using MicroTom as the transformation system. Many re-sequencing studies have indicated considerable sequence diversity among cultivar/accession genomes [4, 12–14] and the newly developed pan-genome strategy revealed the existence of specific genes only belonging to certain cultivars/accessions [11, 15, 16]. Such observations suggest that the different genetic background between the reference genome and others might complicate experiments, e.g. in cloning target genes, potentially leading to failure of the entire experimental design. Therefore, the availability of a high-quality MicroTom genome was badly needed.

In this study, we provide a high-quality genome of MicroTom and conducted comparative genomic analysis with the previously published Heinz tomato genome. Additionally, together with large amounts of RNA-seq data obtained in this study and collected from public databases, we present the RNAome landscape of MicroTom across different organ/developmental stages/treatments and performed comprehensive analyses of the transcriptome of MicroTom protein-coding genes, with respect to (aspects of) gene expression and alternative splicing (AS). With the reference genome and abundant gene expression data, we constructed co-expression networks for tomato organ development, which will provide valuable information for the regulation of organ development in the life cycle. Non-coding RNAs were also identified by combining different sequence strategies, and their interaction networks were predicted. Finally, we constructed a database (MicroTomBase, http://eplant.njau.edu.cn/microTomBase), available for data download, online searching, and demonstration of analytical results. This database will be invaluable for researchers who employ the MicroTom tomato as the laboratory model plant.

## Results

### Assembly of the MicroTom genome and comparative genomics between MicroTom and Heinz

Using a combination of 92.40 Gb Nanopore data and 54.38 Gb Illumina data, we first obtained a MicroTom genome assembly of 799 Mb with 60 contigs (contig N50 = 41.37 Mb). Then, using the genome of Heinz as a reference, 58 MicroTom contigs were further merged to 12 corresponding pseudochromosomes. Protein-coding gene annotation of the MicroTom assembly captures 98.57% of the Embryophyta BUSCO (odb10) genes, with 97.89% single-copy genes and 0.68% duplicates. These results indicate that our assembled MicroTom genome has reached a high standard of quality and completeness (Table 1).

**Table 1:** Statistics and comparison of MicroTom and Heinz genomes

	**MicroTom**				**Heinz 1706 (SL4.0)**			
	**Contigs**		**Pseudomolecules**		**Contigs**		**Pseudomolecules**	
	Size (bp)	Number	Size (bp)	Number	Size (bp)	Number	Size (bp)	Number
N50	41 367 923	8	66 788 879	6	6 007 830	37	65 269 487	6
Maximum length	68 578 620		94 893 795		26 291 688		90 863 682	
Total number	60		12		448		12	
Gap number	47				435			
Assembled length	798 935 606				782 475 302			
BUSCO completeness	98.57%				97.40%			
Gene model number	35 213				34 690			

**Figure 1:**
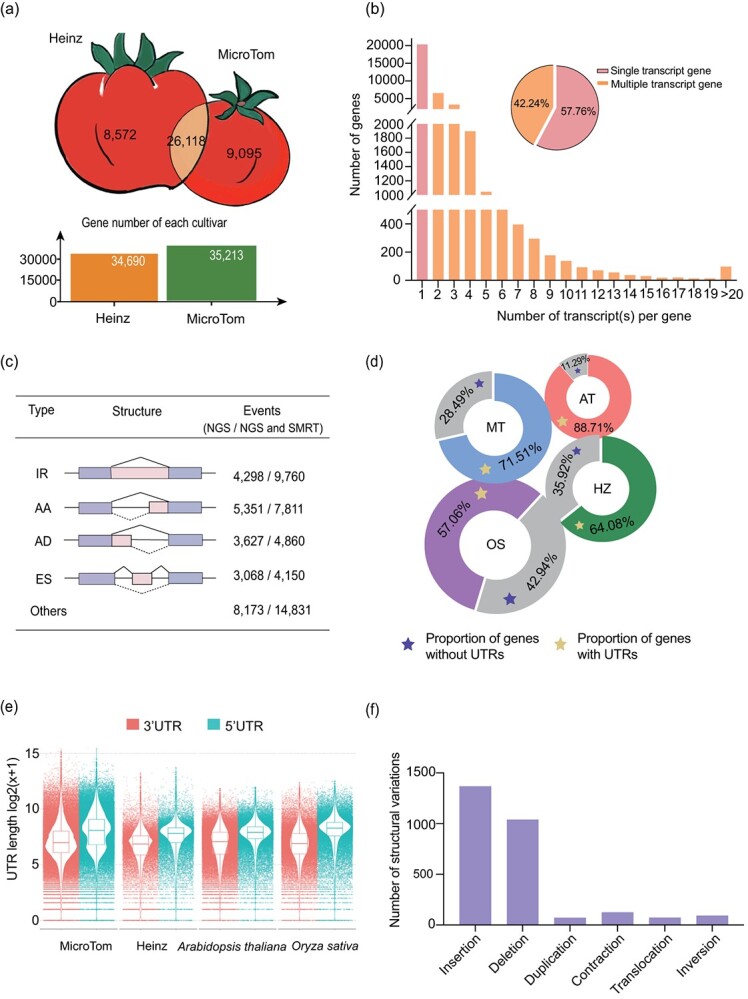
Genome annotation of MicroTom and comparison with Heinz and other species. **a** Protein-coding gene numbers for Heinz and MicroTom varieties. Shared one-to-one orthologs in MicroTom and Heinz genomes are presented in the cross-section of the two tomato cartoon pictures. **b** Proportion of genes with multiple transcripts and genes with different transcript numbers. **c** Categories and frequency of AS events in MicroTom. IR, intron retention; AA, alternative adaptor; AD, alternative donor; ES, exon skipping. **d** Proportion of genes with annotated UTRs in MicroTom (MT), Heinz (HZ), *Arabidopsis thaliana* (AT), and *Oryza sativa* (OS). **e** Dot plot of annotated UTR lengths in MicroTom, Heinz, *A. thaliana* and *O. sativa*. **f** Detected structural variation events of MicroTom compared with Heinz. The minimum insertion, deletion, duplication, and contraction size is 150 bp, the minimum inversion size is 1 kb, and the minimum translocation size is 10 kb.

With the assistance of three full-length transcriptome RNA-seq, 12 ribo-minus RNA-seq data, and 69 poly-A-enriched RNA-seq datasets derived from different organs/tissues under different developmental stages and/or treatments (see Materials and methods) from this and previous studies [3, 17], 35 213 protein-coding genes were annotated in MicroTom. Among these, 31 891 genes (90.57%) received transcriptomic data support from at least one RNA-seq sample. Although MicroTom has a similar number of protein-coding genes to Heinz (34 690), these two cultivars share only 2/3 one-to-one orthologous genes according to synteny analysis and bi-directional BLAST (Fig. 1a), yet each cultivar encodes thousands of specific genes, comprising real specific genes, paralogs by duplications, and genes missed by annotation (mainly in Heinz, probably due to inadequate RNA-seq data) (Supplementary Data Fig. S1). For the two cultivars, phylogenetic trees indicated that MicroTom was sister to Heinz, and wgd analysis indicated that the two cultivars shared the same polyploidy events (Supplementary Data Fig. S4, Supplementary Data Fig. S5, Supplementary Data Fig. S6). However, 4449 MicroTom genes were ‘hiding’ in the Heinz genome (gene length coverage and DNA sequence identity >90%), i.e. not annotated, while the corresponding number of Heinz genes not annotated in MicroTom was about half of 4449. This comparison indicates that adequate and diverse transcriptomic data are critical to a more comprehensive genome annotation.

While AS has been widely detected in eukaryotes to generate functionally divergent transcripts from a common parental gene [18], the Heinz genome did not provide multi-transcript annotation for protein-coding genes. By incorporating dozens of RNA-seq datasets from PacBio and Illumina platforms, 14 873 (42.24%) MicroTom protein-coding genes were identified to have multiple transcripts (Fig. 1b), with an average of two transcripts per gene. Among all AS transcripts, intron retention accounts for the largest proportion (23.57%), followed by alternative acceptor (18.86%) and alternative donor (11.74%) (Fig. 1c). The expression patterns of all transcripts were examined based on the transcriptomic data (Supplementary Data Table S1). Among the AS transcripts, the majority (>70%) showed obvious differential expression in different organs and/or developmental stages (Supplementary Data Table S1), suggesting that these AS transcripts may be functionally distinct and regulate organ differentiation and development in a more prevalent manner than we previously thought.

Due to the large amount of transcriptomic data, more comprehensive information for the untranslated regions (UTRs) was also available. About 71.28% of MicroTom genes have annotated UTRs, showing an obviously higher percentage than that in the Heinz and rice genomes (Fig. 1d), and the median lengths of 5′ and 3′ UTRs are 125 and 271 bp, respectively (Fig. 1e). The annotation of UTRs should provide useful information for gene regulation and expression studies.

Comparing the genomes of MicroTom and Heinz, >1 000 000 single-nucleotide polymorphism (SNP) sites and 40 000 insertion–deletions (indels) (<150 bp) were detected. Additionally, a great number of larger structural variations were also identified (Fig. 1f), such as bigger-sized genomic insertions, deletions, duplications, contractions, translocations, and inversions. These small and large structural variations indicate genomic divergence between the two tomato cultivars, which may affect experimental results that demand high accuracy, e.g. SNPs and indels in the coding regions would greatly affect the design of CRISPR guide RNAs.

### Co-expression networks

**Figure 2:**
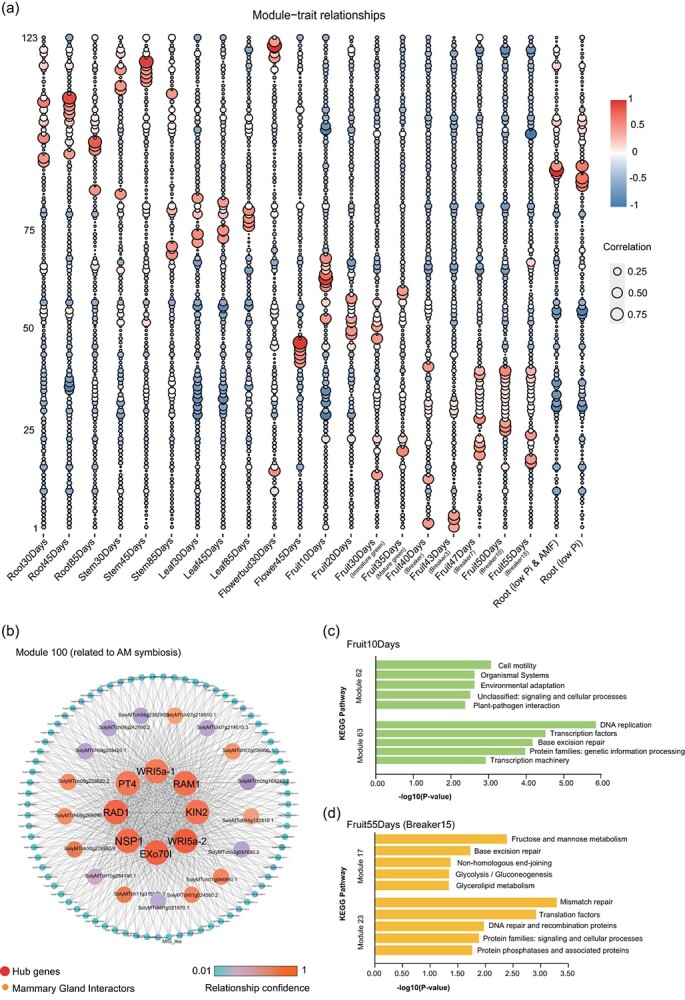
MicroTom co-expression networks established based on 22 transcriptome samples of different organs/developmental stages/treatments. **a** One hundred and twenty-three modules and their correlation with 22 phenotypes or molecular processes. **b** Module 100 and its network, significantly correlated with AM symbiosis. Gene names (instead of IDs) indicate that these genes have been functionally characterized and are involved in AM symbiosis. Threads indicate correlation between genes. **c** Top five enriched KEGG pathways in the early stage of fruit development (10 days after anthesis; Fruit10Days in panel **a**) in the most correlated modules 62 and 63. **d** Top five enriched KEGG pathways in the fully mature stage of fruit development [55 days after anthesis Fruit55Days (Breaker15) in panel **a**] in the most correlated modules 17 and 23.

Co-expression networks underlying the development of organs and response to stimuli were inferred using transcriptome data derived from 22 different tissues/organs and at different developmental stages and/or different treatments (three replicates for each sample). All expressed genes were classified into 123 modules (Fig. 2a, Supplementary Data Fig. S2, Supplementary Data Table S2), whereby each module contains genes that share a similar expression pattern, and possibly function in the same regulatory network, defining and/or regulating a specific phenotype. When a specific phenotype is defined, the corresponding module can be identified, and the hub genes, as well as the entire network linked to the phenotype, can be extracted. This way, unknown genes in the pathway can be identified, potentially providing important clues of targets and gene interactions.

For instance, our results indicate that the AM symbiosis in MicroTom (root, low phosphorus, and arbuscular mycorrhizal fungi) is significantly associated with module 100 (Fig. 2a and b, Supplementary Data Table S2). It could be noticed that several important genes for known functions in the AM symbiosis pathway are present, and the majority of them are even hub genes in the network, including PT4, NSP1, RAD1, RAM1, Exo70I, KIN2, and two WRI5a orthologs (designated WRI5a-1 and WRI5a-2). It is well known that RAM1, RAD1, and NSP1 are regulators relatively upstream in the AM symbiosis pathway [19–23], and WRI5a is even considered as a master regulator [24], so they should have broad associations with other genes in the same pathway. Therefore, this co-expression result makes good sense, not only in identifying the right (expected) genes, but likely also in suggesting other critical, potentially even hub, genes. In this case, other unknown genes in the pathway can also be identified through such expression association (guilt by association), and co-expression analysis should serve as an effective means to explore unknown functional genes in pathways.

Tomato is one of the most important model plants, if not the most important plant, for fruit development studies. Therefore, we collected transcriptomes derived from fruits of nine different development stages (10, 20, 30, 35, 40, 43, 47, 50, and 55 days after anthesis with three replicates each), and observed fruits at different development stages showing specific associations with different transcriptional modules (Fig. 2a). For instance, fruits 10 days after anthesis are associated with modules 62 and 63 (Fig. 2a, Supplementary Data Table S2), the genes of which are functionally enriched in the categories of ‘Cell motility’, ‘Organismal systems’, ‘DNA replication’, and ‘Transcription factors’, suggesting that early development of tomato fruits mainly involves cell division, cell proliferation, and regulatory network development of this organ (Fig. 2c), whereas fruits 55 days after anthesis are associated with modules 17 and 23 (Fig. 2a, Supplementary Data Table S2), the genes of which are functionally enriched in the categories ‘Fructose and mannose metabolism’, ‘Base excision repair’, ‘Mismatch repair’, and ‘Translation factors’, suggesting biosynthesis of metabolites, which mainly seems to occur in fully mature fruits (Fig. 2d). These different modules indicate distinct major regulatory priorities at different developmental stages of tomato fruits.

### Diverse non-coding RNAs, their targets and interactions

**Figure 3:**
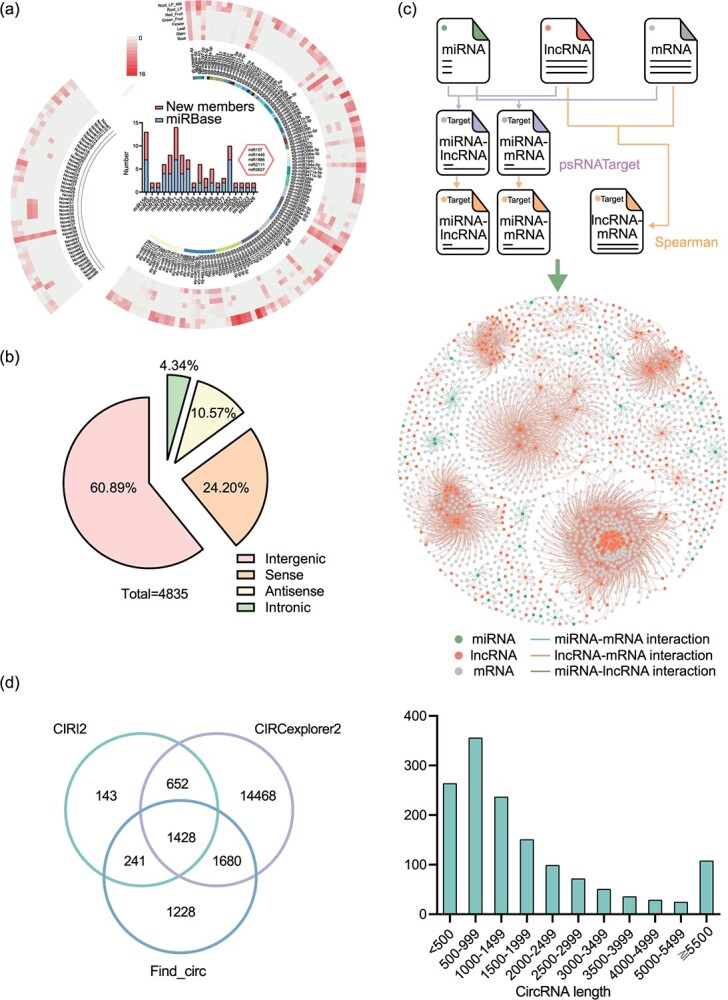
Non-coding RNAs and interaction networks in the MicroTom genome. **a** Identification of MicroTom miRNAs. The right panel represents known miRNA families and their members identified in this study and their expression, and the left panel represents novel miRNA families. The circular heat map shows miRNA expression (outer to inner): 1, roots symbiotic with AM fungi for 6 weeks under low phosphorus condition; 2, roots without AM fungi under low phosphorus condition; 3, red fruits; 4, green fruits; 5, flowers; 6, leaves; 7, stems; 8, roots under normal phosphorus condition. The histogram represents members identified in the known miRNA families of MicroTom, and divides into two parts. One is the newly discovered members filled with red color, another is the existing members of miRBase filled with blue color. The hexagon represents miRNA families previously not identified in tomatoes but identified in other plants. **b** Proportion of four different types of lncRNAs. **c** An integrated interaction network between miRNAs, lncRNAs, and mRNAs. **d** Venn diagram representing the comparison of circRNAs predicted using three softwares. **e** Distribution of circRNA lengths.

Non-coding RNAs, including miRNA, lncRNA, and circRNA, were comprehensively annotated using multiple RNA-seq data sets. These datasets were generated from six development stages/organs of MicroTom tomato plants (root, stem, leaf, flower, green fruit, and red fruit), and under phosphorus deficiency and AM symbiosis status. A total of 210 miRNAs were identified from the sRNA datasets, of which 164 belong to 48 known tomato miRNA families in miRBase (Fig. 3a). We identified 19 tomato miRNA families previously documented in the miRBase with increased family sizes, with one to seven more members discovered for each family. Notably, five miRNA families that have not been documented in tomato (only documented in other plants) were identified in this study, namely miR157, miR1446, miR1886, miR2111, and miR3627. Additionally, 46 novel miRNAs were identified (Fig. 3a). The lengths of MicroTom miRNAs range from 20 to 24 nt, with 21-nt miRNAs accounting for the largest proportion. Expression analysis revealed that most of the identified miRNAs were organ-/developmental stage-specific or were only expressed upon stimulation by Pi deficiency and/or AM symbiosis. Only a few of them could be detected in multiple samples, e.g. miR403-3p, miR9472-5p, and miR166a/g.

Altogether, 4835 lncRNAs were annotated from MicroTom transcripts, and >2944 of them (60.89%) were transcribed from intergenic regions, whereas 720, 511, and 210 lncRNAs were transcribed from the genomic regions overlapping with protein-coding genes at the sense/antisense strand, or from the intronic sequences, respectively (Fig. 3b). The length of MicroTom lncRNAs ranged from 200 to 15 073 nt, with an average length of 986 nt. Similar to miRNAs, the majority of lncRNAs showed development stage-/tissue-specific or stimulus-induced expression. To explore the potential regulatory roles of miRNAs and lncRNAs, pairwise interaction analyses were performed for combinations of miRNA–mRNA, miRNA–lncRNA, and lncRNA–mRNA. The results showed that 266 mRNAs and 7 lncRNAs could be predicted as targets of miRNAs with a significant negative expression correlation. Significant expression correlation was also observed for 8672 lncRNA–mRNA pairs, suggesting potential regulatory relationships. Putting these predicted interactions together, networks integrating miRNAs, lncRNAs, and mRNAs were constructed (Fig. 3c, Supplementary Data Table S3), and these networks should serve as a basic resource for elucidating the regulatory roles of miRNAs and lncRNAs in tomato development and response to stimuli.

A comprehensive annotation of circRNA was performed by incorporating the sequencing results from this study and our previous study [17]. A total of 19 840 circRNAs supported by three independent software tools were identified by mapping the sequencing reads onto the MicroTom genome. The lengths of 1428 identified high-confidence circRNAs ranged from 197 to 27 820 nt, with an average of 1428 nt.

### Construction of MicroTom genomic and RNAomic database

**Figure 4:**
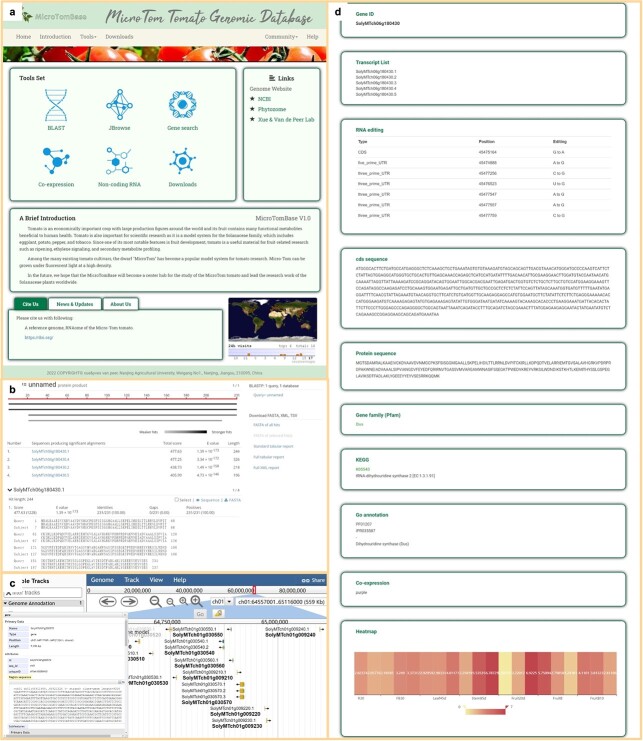
Demonstration of the database MicroTomBase. **a** Home page of MicroTomBase and the main functions, providing service/information of sequence blast, genomic position anchoring, gene ID search, co-expression modules, non-coding RNAs, and data download. **b** The BLAST tool of MicroTomBase. Users can input genomic, PEP, or CDS sequences as the query sequence and the resulting alignment scores are ranked from high to low. **c** The JBrowse tool for visualization of MicroTom genomic details, including gene visualization interface and detailed data on individual genes. **d** Detailed information about genes, including protein and CDS sequence, RNA editing sites, KEGG and GO annotation, gene family, expression profile, and co-expression module.

To make our MicroTom genomic data and analytical results conveniently accessible, we constructed a database (MicroTomBase, http://eplant.njau.edu.cn/microTomBase), providing online search and download possibilities for our data and results (Fig. 4). Through gene ID or sequence BLAST searches, interesting genes can be found, together with detailed information, including genomic positions, gene sequence with detailed structure information [UTRs and coding sequences (CDSs)], functional annotation, multi-transcripts, and expression profiles. Information on non-coding RNAs and their targets as well as interacting networks is also provided under the ‘Non-coding RNA’ section, which can also be accessed through BLAST search. All MicroTom protein-coding genes are ascribed to 123 co-expression modules and can be found under the ‘Co-expression’ section. Genome assemblies and annotation files of the MicroTom tomato are available for download. The MicroTom database will facilitate comparative genomics, transcriptional regulation, and functional studies for tomatoes.

## Discussion

Tomato has been playing an increasingly important role as a model plant for studies of fruit development, plant disease resistance, and symbiosis. Despite being the most employed cultivar, and the largest amount and a wide diversity of multi-omic data being available, until now MicroTom tomato has lacked a high-quality genome. This study fills this gap by providing a high-quality MicroTom genome assembly, obtained by a combination of Nanopore and Illumina sequencing. Moreover, we integrated a diversity of transcriptome data (obtained from both public resources and our own data) to achieve a comprehensive annotation and profound analyses for this genome, reporting more genes, more and longer UTRs, multiple AS transcripts, co-expression networks, non-coding RNAs, and their interaction networks, results which should serve as a great resource for further genetic and functional studies.

AS is considered a mechanism to generate multiple transcripts with different functions [18], and a number of studies have reported its extensiveness in organismal life cycles, e.g. organ differentiation and development [25, 26], disease ontogenesis [27, 28], and response to abiotic and biotic stimuli [29–31]. However, the Heinz tomato genome did not provide information for AS. The MicroTom genome annotation fills this gap by including multiple transcripts generated by AS. Over 40% of MicroTom genes are alternatively spliced and the majority of AS transcripts show differential expression under different conditions, suggesting a broad functional role of AS.

Other than assisting in-depth genome annotation, the large amounts of transcriptomes also provide data for our co-expression network analyses, which classified MicroTom transcripts into different expressional modules, and accordingly established diverse networks associated with different phenotypes (different organs, developmental stages and treatments). Potential genes with important functions that have not been recognized can thus be identified based on their association and applied for further functional characterization. Therefore, our co-expression networks should provide useful information for a more precise targeting of candidate genes related to specific phenotypes in the tomato life cycle.

Non-coding RNAs are extensively involved in the post-transcriptional regulation of gene expression through different mechanisms, and play important roles in a variety of life processes in plant growth and development, stress resistance, and interaction with pathogenic or beneficial microbes [32, 33]. In tomato, high-throughput sequencing of miRNAs of MicroTom have been reported [17, 34–36], and functional roles of miRNAs in fruit developmental regulation have been characterized, e.g. miRNA156 [37] and miRNA159 [38]. The profiles of lncRNAs and circRNAs of MicroTom have also been investigated, but by only a few studies [17, 39]. Moreover, these studies merely focused on non-coding RNAs at certain specific developmental stages or treatments, while a full picture of tomato non-coding RNAs is still lacking. In this study, we comprehensively annotated miRNAs, lncRNAs, and circRNAs in the MicroTom genome by integrating multiple datasets generated from diverse organs, developmental stages and treatments (Supplementary Data Table S3), and our results greatly extended the non-coding RNA list of tomato. Furthermore, the expression profile of non-coding RNAs among different samples indicate that most of the identified miRNAs and lncRNAs were specifically expressed in different organs and developmental stages or under AM symbiosis, suggesting a conditional induction of these non-coding RNAs. Furthermore, non-coding RNAs can regulate the expression of protein-coding genes, directly or coordinately affecting the translation and homeostasis of mRNAs [40]. By constructing the interaction networks of the MicroTom miRNAs, lncRNAs, and mRNAs, we uncovered highly complicated regulatory relationships between non-coding RNAs and their target mRNAs, which provides novel insights into the post-transcriptional regulation of protein-coding genes in MicroTom.

In summary, this study presents a high-quality genome for the MicroTom tomato, and a comprehensive annotation for both coding and non-coding genes, including splice variants and UTRs. Taking advantage of large amounts of transcriptomes, the gene expression profile and co-expression networks are constructed, which will provide clues for the identification of novel genes involved in diverse pathways. Non-coding RNAs, including miRNAs, lncRNAs, and circRNAs, as well as their potential targets, were identified and interacting networks were constructed, which will provide valuable information for future studies dedicated to exploring the post-transcriptional regulation of plant development by non-coding RNAs. All these findings suggest diverse and complicated modifications and regulations at the RNA level. All these results have been integrated into our online resource, which we hope could provide an invaluable resource for researchers studying tomatoes and employing tomato as a model plant.

## Materials and methods

### Plant material and data sources

Seedlings of *S. lycopersicum* (cv. MicroTom) were grown in plastic pots filled with a mixture of sterilized sand/gravel (1:1 ratio) in a climate-controlled growth room with 16 h light at 24°C and 8 h dark at 22°C. Roots, stems, leaves, flowers, immature green fruits, and mature red fruits were collected from plants grown in nutrient-rich soil for 6 weeks, and used for RNAome sequencing. Green and red fruits were classified using the USDA Visual Aid TM-L-1 color chart (USDA, Agricultural Marketing Service, 1975).

We also downloaded 75 Illumina RNA-seq datasets and two PacBio RNA-seq datasets involving different tissues/organs and different developmental stages and/or different treatments from our previous study [17], and a study performed by others [3]. Twelve MicroTom tomato proteomic datasets were retrieved from the PRIDE Archive database (Proteomics Identifications Database, https://www.ebi.ac.uk/pride/archive/).

### Library construction and sequencing

Fresh leaves of 4-week-old MicroTom that were grown in half-strength Murashige and Skoog basal medium with sucrose and Phytagel were collected, and the genomic DNA was sequenced on the Nanopore platform using a MinION R9 flow cell and the Illumina NovaSeq6000 platform with an insert size of 450 bp at Benagen (Wuhan, China).

Fresh tissues of roots, stems, leaves, flower, immature green fruits, and mature red fruits of plants grown in nutrient-rich soil were subjected to total RNA extraction using TRIzol (Invitrogen, Carlsbad, USA) according to the manufacturer’s protocol, and were used for subsequent RNA sequencing using different strategies. Samples from each tissue were subjected to construct a ribo-minus RNA library and small RNA library for RNA sequencing, whereas a mixture of the six samples was used to construction a PacBio SMRT library and a circular RNA library. The ribo-minus RNA libraries, small RNA libraries, and circular RNA library were sequenced using the Illumina HiSeq platform at Novogene Co., Ltd (Tianjin, China), and the PacBio SMRT library was sequenced using the PacBio Sequel System at Novogene Co., Ltd (Tianjin, China).

### Genome assembly and assessment

Firstly, the raw Nanopore reads were corrected and assembled into contigs by NextDenovo-v2.5.0 (https://github.com/Nextomics/NextDenovo) with default parameters. Then, Nanopore reads were further mapped into primary contigs by the software Minimap2 v2.17 [41] with the parameter -ax map-ont, followed by Nextpolish v1.4.1 [42] to polish contigs. Meanwhile, the Illumina paired-end reads were processed to remove adaptor and low-quality sequences using Trimmomatic v0.38 [43]. Then, the clean Illumina short reads were mapped to the polished contigs using BWA-MEM v0.7.17 [44] with default parameters, based on three iterative rounds of polishing with parameter —fix all by Pilon v1.23 [45] (read length N50 = 41.37 Mb). Finally, the polished contigs were aligned to the Heinz genome to form pseudomolecules, by the software RaGOO v1.1 [46]. Embryophyta BUSCO v5.4.2 (odb10) [47] was used to evaluate the integrity of the final assembly.

### PacBio transcriptome processing

The SMRTlink v6.0.0 pipeline was applied to PacBio *lso-seq* raw data. Firstly, the circular consensus sequence reads (CCSs) were extracted from subreads of BAM files with parameters minLength = 300, minZScore = −999, minPasses = 1, maxDropFraction = 0.8, minPredictedAccuracy = 0.8, minSnr = 4, noPolish = TURE. Secondly, CCS reads were divided into full-length non-chimeric (FLNC) reads and non-full-length (nFL) reads by identifying whether 5′ and 3′ adapters and the poly(A) tail were present. Then, we obtained consensus isoforms using the ICE (Iterative Clustering for Error Correction) algorithm from FLNC reads, which were further polished with nFL reads to get high-quality consensus FLNC reads based on Quiver (parameters hq_quiver_min_accuracy 0.99; others with default parameters). Finally, the non-redundant FLNC reads were obtained by CD HIT v4.8.1 [48] with an identity cutoff of 0.95. We also used the software LoRDEC v0.9 [49] to correct mismatch and nucleotide indels in FLNC reads.

### Repetitive DNA identification and genome annotation

We identified the repetitive elements in the MicroTom genome based on a combination of *de novo* and homology-based strategies. A *de novo* library was first built through RepeatModeler v2.0.1 [50] and LTR_retriever v2.9.0 [51]. The repeat region in the genome was further masked by scanning the *de novo* repeat library. Next, we used RepeatMasker v4.0.9 [52] to classify the repeat sequences using the parameters -poly -html -gff -lib -no_is -xsmall.

The combination of *ab initio*, homology-based and RNA-seq-based annotation strategy was applied to predict and annotate the protein-coding genes. Firstly, the masked genome was trained by BRAKER2 v2.1.5 [53] and GeneMark-ET v4.69 [54] for *ab initio* gene prediction. Secondly, to find homologous genes, related species like *Solanum tuberosum* (686_v6.1) and *S. lycopersicum* Heinz 1706 (691_ITAG4.0), which were downloaded from Phytozome v13, were loaded into GenomeThreader v1.7.1 [55] with the parameters -species arabidopsis -gff3out -intermediate. Thirdly, 81 RNA-seq (Supplementary Data Fig. S3, Supplementary Data Table S4) data sets were used to assist the annotation. These were evaluated by FastQC v0.11.9 and Trimmomatic v0.38 [43] to trim low-quality and adaptor sequences. Then, these high-quality reads were aligned to the genome by HISAT2 v2.2.1 [56]. We assembled transcripts with StringTie v2.1.5 [57] and TransDecoder v5.5.0 (https://github.com/TransDecoder/TransDecoder). The PacBio *lso-seq* was also applied to forecast complete CDSs by the PASA v2.5.2 pipeline [58]. Finally, the EVidenceModeler v1.1.1 pipeline was applied to integrate the above-mentioned prediction strategies [59]. The GTF annotation file, assembled from the Illumina and PacBio data, was used for identifying AS events using the AStalavista v3.2 tool [60].

### Functional annotation

Several public databases (PFAM, GO, and KEGG) were used to map GO terms for searching functional motifs and domains by InterProScan v5.50–84.0 [61]. When the gene ontology terms for each gene were obtained, functions of protein-coding genes in the MicroTom genome were annotated by the Swiss-Prot database, only retaining the best alignment.

### Identification of structural variations and SNPs between reference genomes

Structural variations (SVs) were identified by genome-wide alignment between the MicroTom and Heinz 1706 (ITAG4.0) genomes using MUMandCo v3.8 [62], which can detect over 1 kb structural variations in length. We also used the MUMmer package v4.0.0 [63] to align these two genomes. The reciprocal best alignments were found using the delta-filter program. Then, the uniquely aligned fragments were used to identify SNP sites and indels with the show-snp tool.

### Gene expression and construction of co-expression networks

The RNA-seq dataset for gene co-expression analysis was obtained from samples with 22 various tissues/organs and at different developmental stages and/or different treatments. The normalized expression of RNA-seq data was calculated with FPKM (fragments per kilobase per million reads) from all samples. FPKM values <1 were filtered out. We used the R package WGCNA v1.66 [64] to construct co-expression networks, and the soft threshold value was calculated by pickSoftThreshold integrated in WGCNA. We used cytoHubba and MCODE to identify candidate hub genes, and used Cytoscape v3.9.1 [65] to visualize the co-expression networks.

### Identification and annotation of non-coding RNAs

The prediction of protein-coding potential for transcripts generated from Illumina and SMRT data was conducted by integrating CPC2 v0.1, CNCI v2, PLEK v1.2, and PFAM v32 [66–69]. Candidate lncRNAs were defined as transcripts with the longest representative sequence lacking any open reading frame (ORF) exceeding 100 amino acids, while having a minimum nucleotide sequence length of 200 nt. MiRNAs were identified using miRador (https://github.com/rkweku/miRador) with slight modification [70]. Specifically, the sequences with the highest abundance of each miRNA locus were added as candidate miRNAs. Candidate miRNAs were annotated as known or novel miRNAs through referencing the latest miRBase (v22.1) [71]. CIRI2 v2.0.6, CIRIexplorer2 v2.4.0, and Find_ciric v1.2 were combined to detect potential back-splice sites of candidate circRNAs [72–74]. Reconstruction of full-length circRNAs was achieved using CIRI-full [75].

### Identification of targets of miRNAs/lncRNAs

Potential miRNA targets (mRNAs and lncRNAs) were firstly predicted by using the psRNATarget v2 program. Then, the expression values of resulting miRNA–mRNA/lncRNA pairs for six tissues (roots, stems, leaves, flower, immature green fruits, and mature red fruits) were subjected to Spearman correlation coefficient analysis. MiRNA–mRNA/lncRNA pairs were considered to be a candidate interactional miRNA target when the Spearman correlation coefficient was <−.80 and the *P* value was <.01.

To predict lncRNA–mRNA co-expressed pairs, Spearman correlation analysis was performed between lncRNAs and mRNAs in 75 RNA-seq samples. For more accurate prediction of lncRNA–mRNA correlations, we increased the threshold of the Spearman correlation coefficient to >.9 and the *P* value <.01. We also screened the results that met the above requirements based on six or more samples with FPKM >1, considering these lncRNA–mRNA pairs to be co-expressed.

## Acknowledgements

This work was supported by grants from the Fundamental Research Funds for the Central Universities (KYCXJC2022003), the National Natural Science Foundation of China (32070243), and the Outstanding Young Teacher of the QingLan Project of Jiangsu Province. Y.V.d.P. acknowledges funding from the European Research Council (ERC) under the European Union’s Horizon 2020 research and innovation program (833522) and from Ghent University (Methusalem funding, BOF.MET.2021.0005.01). The computational resources and services were provided by the Bioinformatics Center of Nanjing Agricultural University.

## Author contributions

J.Y.X., Z.Q.S., F.C., and Y.V.d.P. conceived the study. Z.Q.S. and Z.Z. collected plant samples. H.Y.F. conducted genome assembly and annotation. H.Y.F., Z.Z., S.Y.H., Y.J.C., X.R.M., and S.X.L. conducted analyses of RNA-seq data. Y.H.Z. constructed the database. J.Y.X. and Z.Q.S. drafted the manuscript. All authors read the manuscript and participated in the revision of the manuscript. All authors approved the final manuscript.

## Data availability

The data supporting the findings of this work are available within the paper and its supplementary information files. The datasets generated and analyzed during this study are available from the corresponding author upon request. All sequencing data generated have been deposited in National Genomics Data Center (https://bigd.big.ac.cn/) with BioProject ID PRJCA012325 under GSA CRA008482. The genome assembly, along with the gene models, the functional annotation, and some supplementary information files are available on FigShare at the link: (https://doi.org/10.6084/m9.figshare.21509817). All this information can also be accessed under GWH: WGS029763 and MicroTomBase (http://eplant.njau.edu.cn/microTomBase).

## Conflict of interest

The authors declare that the research was conducted in the absence of any commercial or financial relationships that could be construed as a potential conflict of interest.

## Supplementary data

Supplementary data is available at *Horticulture Research* online.

## Supplementary Material

Web_Material_uhad147
